# Sexual orientation identity in relation to unhealthy body mass index: individual participant data meta-analysis of 93 429 individuals from 12 UK health surveys

**DOI:** 10.1093/pubmed/fdy224

**Published:** 2019-02-21

**Authors:** J Semlyen, T J Curtis, J Varney

**Affiliations:** 1 Norwich Medical School, University of East Anglia, Norwich Research Park, Norwich, UK; 2 Centre for Population Research in Sexual Health and HIV, Institute for Global Health, University College London, London, UK; 3 Public Health England, London, UK

**Keywords:** body mass index, obesity, sexual orientation, social determinants, underweight

## Abstract

**Background:**

Lesbian, gay and bisexual adults are more likely than heterosexual adults to experience worse health outcomes. Despite increasing public health interest in the importance of maintaining a healthy body weight, no study has considered sexual orientation identity (SOI) and unhealthy BMI categories among adults in the UK population.

**Methods:**

Individual participant data meta-analysis using pooled data from population health surveys reporting on 93 429 adults with data on SOI, BMI and study covariates.

**Results:**

Adjusting for covariates and allowing for between-study variation, women identifying as lesbian (OR = 1.41, 95% CI: 1.16, 1.72) or bisexual (OR = 1.24, 95% CI: 1.03, 1.48) were at increased risk of overweight/obesity compared to heterosexual women, but men identifying as gay were at decreased risk (OR = 0.72, 95% CI: 0.61, 0.85) compared to heterosexual men. Increased risk of being underweight was seen for women identifying as ‘other’ (OR = 1.95, 95% CI: 1.07, 3.56), and men identifying as gay (OR = 3.12, 95% CI: 1.83, 5.38), bisexual (OR = 2.30, 95% CI: 1.17, 4.52), ‘other’ (OR = 3.95, 95% CI: 1.85, 8.42).

**Conclusions:**

The emerging picture of health disparities in this population, along with well documented discrimination, indicate that sexual orientation should be considered as a social determinant of health.

## Introduction

Overweight and obesity are strong risk factors for a number of health-damaging conditions including coronary heart disease, stroke, cancer and early mortality.^1–4^ Moreover there is a growing evidence base demonstrating that underweight is also linked to excess mortality.^3,5^ Disparities in unhealthy weight then place those with higher prevalence of unhealthy weight at higher risk of loss of healthy years lived^6^ and reduced life expectancy estimates.^7^

Several subgroups of the population have been identified as having increased risk for overweight and obesity, and policy makers and clinicians have used this evidence to design public health interventions and tailored advice.^8^ For example, National Institute for Health and Care Excellence (NICE) guidelines for obesity identification and management explicitly mention ethnicity and lower socioeconomic status as special groups for targeted interventions.^9^ For sexual minority groups in the UK, there has been a very limited evidence base on which to develop similar interventions.

Until 2008, sexual orientation identity (SOI) was not recorded in UK population health surveys, thus no data from population health surveys was available.^10,11^ This lack of data has serious implications for health disparities for adults who do not identify as heterosexual and has meant that until recently the inequalities affecting lesbian, gay and bisexual (LGB) populations have been hard to analyse at scale through routine datasets.^12,13^

International research has found that sexual minority women are more likely to have an unhealthy body weight (obese or overweight) than heterosexual women,^14–16^ with lesbian identity being associated with greatest levels of obesity.^17^ This association has been found in young,^18^ adult^17^ and older lesbian/bisexual women,^19^ is found within ethnic minority subgroups^20,21^ and is sustained over the life course.^22,23^

Some studies find decreased risk of overweight or obesity in sexual minority males^17,19,20,24,25^ and across the life span,^26^ some report increased risk when comparing bisexual and heterosexual males,^27^ and others no association,^28,29^ but with few population representative samples and considerable heterogeneity in methods across studies, and wide variation in recording sexual identity, there is no consensus of the scale of the risk.

Many studies that look at unhealthy weight in sexual minorities do not include underweight as a category^30^ or exclude underweight as a separate variable in their analysis by combining it with the healthy/normal weight category,^31^ often due to small numbers of respondents,^32,33^ viewing the results as merely showing a reduced prevalence of overweight/obesity. One study that did analyse underweight separately found gay adolescents to be more likely to be underweight than heterosexual adolescents,^28^ but this study did not include sexual minority adult males. In another study, Laska *et al.*^34^ found that bisexual college students were more likely to be underweight than heterosexual college students. By analysing data from only college students in a state-based study in the USA this study has limited generalizability beyond that context.

The aim of our study was to evaluate the association in UK available population level data between SOI, specifically identifying as lesbian, gay, bisexual or ‘other’ (LGBO), and unhealthy body mass index (BMI) defined as either underweight or overweight and obese. This was achieved by pooling individual participant data from 12 studies.^12^ Additionally, as observed in previous research,^31,34,35^ we evaluated whether associations differed for men and women.

## Method

### Design and setting

Participants were drawn from 12 datasets from five British cohort or cross-sectional health survey studies in which sexual orientation was measured consistently. These were the British Cohort Study (BCS70, 2012),^36^ Health Survey for England (annual waves 2011–13),^37^ National Survey of Sexual Attitudes and Lifestyles (Natsal-3, undertaken 2010–12),^38^ Scottish Health Survey (annual waves 2008–13),^39^ and Understanding Society (2011/12 wave).^40^ Studies were identified by searching the UK Data Service (search terms ‘sexual orientation’, ‘gay’, ‘lesbian’, ‘bisexual’, ‘sexuality’) and the published literature. Data were collected using either home visit interviews, self-completion questionnaires, telephone interviews, web surveys or a combination of methods. Details including sampling designs, and inclusion/exclusion criteria for each dataset are available from the UK Data Archive.^41^ Our study population comprised adults with available data on SOI, BMI and covariates. Data were analysed in 2017.

### Participants and materials

For all included datasets, participants were recruited through random or stratified random sampling of their target population. Full details of sampling for Understanding Society are available (https://www.understandingsociety.ac.uk) and for the other surveys, they are available on the UK Data Service website (www.ukdataservice.ac.uk).

### Sexual orientation identity

SOI was recorded in self-completion questionnaires in all included studies using standardized wording recommended by the Office of National Statistics (ONS).^42^Supplementary Table S1 shows the proportion of participants who refused to answer this question, who were excluded from our study.

**Table 1:** Characteristics of study variables (unweighted) comparing underweight and overweight or obese with healthy body mass index (BMI) categories

	Healthy BMI	Underweight BMI		Overweight/Obese BMI		Total (*n* = 93 429)
	(*n* = 34 244)	(*n* = 1634)	*P*	(*n* = 57 551)	*P*	
Age (25/50/75th percentile)	28/42/54	19/27/42	<0.001	39/48/63	<0.001	34/44/60
Male (%)	38.3	31.5	<0.001	48.6	<0.001	44.5
Lesbian/Gay identity (%)	1.3	2.0	0.02	1.1	<0.001	1.2
Bisexual identity (%)	1.0	1.6	0.03	0.9	0.008	0.9
Other identity (%)	0.6	1.3	0.001	0.8	0.001	0.7
Ethnic minority (%)	9.9	17.9	<0.001	6.7	<0.001	8.1
Degree level education (%)	30.0	20.6	<0.001	23.9	<0.001	26.1
Smoker (%)	26.0	37.6	<0.001	20.4	<0.001	22.7
Longstanding illness/disability (%)	28.9	31.3	0.03	42.4	<0.001	37.3
Married/cohabiting (%)	57.6	32.0	<0.001	68.1	<0.001	63.6

*Notes:* Underweight BMI is defined as a BMI value <18.5 kg/m^2^. Healthy BMI is defined as a BMI value in the range 18.5–24.99 kg/m^2^. Overweight BMI is defined as a BMI value in the range 25–29.99 kg/m^2^. Obese BMI is defined as a BMI value ≥30 kg/m^2^. *P* values are for comparisons of each BMI category separately with the healthy BMI category, and are estimated from chi-square tests. The *P* values for difference in age were calculated using *t*-tests.

### Body mass index

The body mass index (BMI) is body weight in kilograms divided by the square of participants’ height in metres.^43^ Heights and weights were measured during nurse visits, with self-reports used for British Cohort Study and Natsal-3 participants and 49% of included Understanding Society participants. BMI values were converted into categories defined by the World Health Organization.^44^

### Covariates

Covariates were selected on the basis that they are known to be associated with SOI and with BMI (i.e. are potential confounding factors). Covariates were harmonized across studies to ensure comparability: age, sex (male or female), ethnic group (White versus ethnic minority), educational attainment (a 5-point scale ranging from ‘none’ to university degree), smoking status (current smoker versus non-smoker), longstanding illness/disability (yes or no) and married or cohabiting (yes or no).

### Statistical analysis

Bivariate associations between SOI categories, BMI categories and covariates were first evaluated using *t*-tests and chi-square statistics. We used an *α* of 0.05 for all statistical tests. For the main analysis, individual participant data (IPD) meta-analysis with logistic regression was used to evaluate the associations between SOI categories and unhealthy BMI categories (underweight, or overweight/obese), adjusting for covariates. IPD meta-analysis enables more flexible and potentially more powerful statistical analyses than are possible with aggregate data. Unlike most meta-analyses, they do not rely on aggregate data extracted from journal publications. Rather, the original data on each individual participant are sought from each eligible study. In this case each eligible study is one of several health surveys that collected data on SOI and BMI. These original data are then used to calculate summary statistics for each study before pooling these estimates, accounting for heterogeneity between studies.^45–48^ A key benefit of IPD meta-analysis is that it may allow the estimation of associations for smaller subgroups (such as individuals identifying as LGBO) for which the original studies were underpowered.

We used data from studies conducted over a range of years and with slightly different UK geographic foci, and so it was reasonable to assume there might be some heterogeneity in true effect sizes across studies, even once other factors were adjusted for. In conducting our meta-analyses we therefore assumed a random effects model (assumes the true effects for individual studies are normally distributed about some average effect). The study-specific odds ratios and their standard errors are pooled to produce an estimate of the average effect size for the studies. We used the Paule–Mandel method to estimate between-study variance.^49^

In preliminary analyses, we found evidence that effects differed significantly for men and women, leading us to separate them for the main analysis (*P* value for interaction < 0.01). In sensitivity analyses, we repeated results after excluding each individual study separately, to evaluate the impact of individual studies on the findings. We also checked whether results differed materially when using the alternative approach to IPD meta-analysis, where all data are analysed simultaneously with a random effect for study of origin. To evaluate the impact of survey design features, we repeated models using Understanding Society and Natsal-3 (the largest contributing studies and with complex survey design) before and after adjustment using sampling weights. All analyses were conducted using Stata version 14.0 (StataCorp. 2015, College Station, TX).

### Ethical approval

For all of the original studies used, ethical approval was provided by a university or local research ethics committee (the UK Data Service website hosts details for each study).

## Results

### Univariate analyses

There were 93 429 adults in the analytic sample (Table 1), with 1095 (1.2%) identifying as lesbian/gay, 873 (0.9%) as bisexual and 675 (0.7%) as ‘other’. Compared to those with a healthy BMI, those with an underweight BMI were significantly younger (median age 27 versus 42 years), comprised a smaller proportion of men (31.5 versus 38.3%), more ethnic minorities (17.9 versus 9.9%), a higher proportion of smokers (37.6 versus 26.0%), and a smaller proportion who were married/cohabiting (32.6 versus 58.4%). Compared to those with a healthy BMI, those with an overweight or obese BMI were significantly older (median age 48 versus 42 years), comprised a larger proportion of men (48.6 versus 38.3%), more longstanding illness/disability (42.4 versus 28.9%) and a higher proportion who were married/cohabiting (68.7 versus 58.4%).

Compared to their heterosexual counterparts, lesbian, gay and bisexual men and women were younger, while men and women identifying as ‘other’ were significantly older (Table 2). More lesbian women and gay men were educated to degree level than heterosexual women and men, while fewer were ethnic minorities. In contrast, a greater proportion of men and women identifying as bisexual or ‘other’ were ethnic minority when compared to heterosexual men and women. In general, fewer heterosexual men and women were smokers, while more were married or cohabitating than men and women identifying as lesbian, gay, bisexual or ‘other’.

**Table 2:** Characteristics of participants identified as lesbian/gay, bisexual and ‘other’ compared to heterosexuals.

Women	Heterosexual	Lesbian		Bisexual		Other	
	(*n* = 50 463)	(*n* = 452)	*P*	(*n* = 530)	*P*	(*n* = 398)	*P*
Underweight BMI (%)	2.1	2.4	0.67	3.0	0.17	3.0	0.023
Overweight or obese BMI (%)	57.0	59.3	0.34	54.2	0.18	65.8	<0.001
Age (25/50/75 percentile)	34/44/59	28/40/46	<0.001	23/30/42	<0.001	39/49/63	<0.001
Ethnic minority (%)	8.0	4.7	0.01	11.5	0.003	19.6	<0.001
Degree level education (%)	25.6	39.4	<0.001	27.4	0.34	10.1	<0.001
Smoker (%)	21.7	33.2	<0.001	34.2	<0.001	26.4	0.03
Longstanding illness/disability (%)	37.9	40.3	0.30	41.5	0.09	44.2	0.01
Married/cohabiting (%)	62.1	54.7	0.001	44.3	<0.001	58.0	0.09

Men	Heterosexual	Gay		Bisexual		Other	
	(*n* = 40 323)	(*n* = 643)	*P*	(*n* = 343)	*P*	(*n* = 277)	*P*
Underweight BMI (%)	1.2	3.4	<0.001	2.9	0.003	3.3	0.002
Overweight or obese BMI (%)	67.5	52.4	<0.001	60.4	0.005	67.9	0.90
Age (25/50/75th percentile)	35/45/61	29/42/48	<0.001	29/42/58	<0.001	37/55/67	<0.001
Ethnic minority (%)	8.0	4.7	0.002	13.7	<0.001	18.4	<0.001
Degree level education (%)	26.6	39.5	<0.001	27.1	0.82	17.3	0.001
Smoker (%)	23.5	31.4	<0.001	30.6	0.002	26.4	0.26
Longstanding illness/disability (%)	36.2	39.5	0.08	44.0	0.003	44.8	0.003
Married/cohabiting (%)	68.3	40.6	<0.001	46.9	<0.001	53.4	<0.001

*Notes*: Underweight BMI is defined as a BMI value <18.5 kg/m^2^. Overweight BMI is defined as a BMI value in the range 25–29.99 kg/m^2^. Obese BMI is defined as a BMI value ≥30 kg/m^2^. *P* values are for comparisons of each sexual orientation identity separately with Heterosexual identity, and are estimated from chi-square tests. The *P* values for difference in age were calculated using *t*-tests.

Characteristics of participants across each study separately are provided in Supplementary Table S1, which additionally shows the individual sample sizes contributing to our study.

### Associations between BMI and sexual orientation identities

**Fig. 1:**
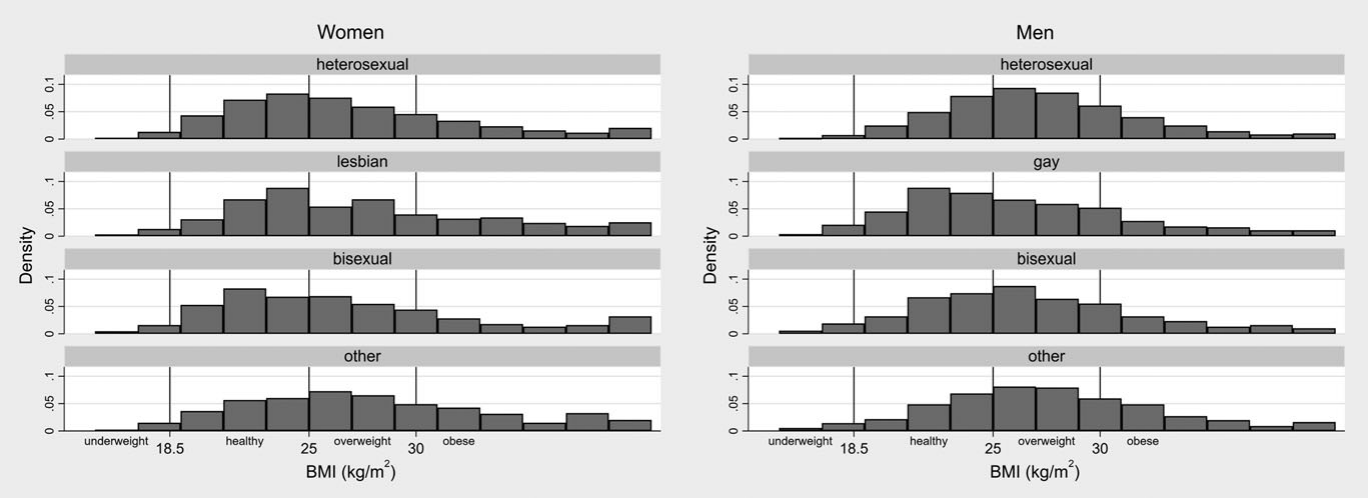
(Histogram). Unweighted distribution of BMI by gender and sexual orientation identity. *Note*: Bin-width equal to 2 kg/m^2^. Those with BMI < 15 kg/m^2^ are grouped in the first bin (15–17). Those with BMI > 41 kg/m^2^ are grouped in the final bin (39–41).

Histograms showing the distribution of BMI for women and men of each SOI category are shown in Fig. 1. The results of the main analyses looking at associations between unhealthy BMI categories and sexual orientation identities are shown in Table 3. Forest plots showing the pooling of results from individual studies are provided in the Supplementary material.

**Table 3:** Odds ratios (95% confidence intervals) for underweight and overweight/obese BMI for women and men

	Underweight BMI			Overweight/Obese BMI		
	% (*n*)	OR (95% CI)		% (*n*)	OR (95% CI)	
		Minimally adjusted^a^	Additionally adjusted^b^		Minimally adjusted^a^	Additionally adjusted^b^
Women						
Heterosexual (*n* = 50 463)	2.1 (1080)	(Reference)	(Reference)	57.0 (28 783)	(Reference)	(Reference)
Lesbian (*n* = 452)	2.4 (11)	1.39 (0.75, 2.60)	1.29 (0.69, 2.40)	59.3 (268)	1.36 (1.12, 1.65)	1.41 (1.16, 1.72)
Bisexual (*n* = 530)	3.0 (16)	1.54 (0.75, 3.18)	1.22 (0.63, 2.39)	54.2 (287)	1.26 (1.05, 1.50)	1.24 (1.03, 1.48)
Other (*n* = 398)	3.0 (12)	2.45 (1.35, 4.43)	1.95 (1.07, 3.56)	65.8 (262)	1.38 (1.05, 1.82)	1.25 (0.96, 1.62)
Men						
Heterosexual (*n* = 40 323)	1.2 (474)	(Reference)	(Reference)	67.5 (27 219)	(Reference)	(Reference)
Gay (*n* = 643)	3.4 (22)	3.27 (2.07, 5.15)	3.12 (1.83, 5.32)	52.4 (337)	0.64 (0.54, 0.75)	0.72 (0.61, 0.85)
Bisexual (*n* = 343)	2.9 (10)	3.07 (1.58, 5.98)	2.30 (1.17, 4.52)	60.3 (207)	0.82 (0.65, 1.03)	0.92 (0.73, 1.17)
Other (*n* = 277)	3.2 (9)	4.92 (2.38, 10.16)	3.95 (1.85, 8.42)	67.9 (188)	0.86 (0.66, 1.12)	0.99 (0.76, 1.30)

*Notes*: Percentages shown are the percentage of participants of each sexual orientation identity with BMI values categorized as either underweight, or overweight or obese. Underweight BMI is defined as a BMI value < 18.5 kg/m^2^. Overweight BMI is defined as a BMI value in the range 25–29.99 kg/m^2^. Obese BMI is defined as a BMI value ≥30 kg/m^2^. ^a^Minimally adjusted for age. ^b^Additionally adjusted for ethnic minority status, educational attainment, cigarette smoking, longstanding illness/disability and relationship status.

After adjustments for a range of covariates, women identifying as lesbian were at an increased risk of being overweight or obese (OR = 1.41, 95% CI: 1.16, 1.72) when compared with heterosexual women, as were women identifying as bisexual (OR = 1.24, 95% CI: 1.03, 1.48). Women identifying as ‘other’ were at an increased risk of underweight BMI (OR = 1.95, 95% CI: 1.07, 3.56), but were not at increased or decreased risk of overweight or obese BMI (OR = 1.28, 95% CI: 0.96, 1.62).

Men identifying as gay were significantly more likely than heterosexual men to be underweight (OR = 3.12, 95% CI 1.83, 5.32), as were men identifying as bisexual (OR = 2.30, 95% CI: 1.17, 4.52) and ‘other’ (OR = 3.95, 95% CI: 1.85, 8.42). Men identifying as gay were significantly less likely than heterosexual men to have an overweight or obese BMI (OR = 0.72, 95% CI: 0.61, 0.85). No associations were found between overweight or obese BMI and bisexual (OR = 0.92, 95% CI: 0.73, 1.17) or ‘other’ (OR = 0.99, 95% CI: 0.76, 1.30) identity in men.

These results are reflected in Fig. 1, which shows the distribution of gay men’s BMI skewed towards lower values, while the distributions of men identifying as bisexual or ‘other’ look similar to that of heterosexual men. Similarly, the distributions for women identifying as lesbian or ‘other’ appear to have heavier tails at the higher end of the BMI range, and both have positively shifted means compared to heterosexual and bisexual women. As no significant association was found for overweight/obese BMI in women identifying as ‘other’, these features instead reflect demographic differences between this group and others.

### Sensitivity analyses

The smaller number of underweight adults in the pooled data means that effects for underweight BMI were more sensitive to removal of individual studies, and confidence intervals for underweight BMI estimates are wider. In the majority of analyses, no significant between-study heterogeneity was found (see Supplementary Table S2), however, results were similar after excluding individual studies where there was evidence of between-study variation. Running the analyses again while artificially setting the between-study heterogeneity at the upper bound of the *I*^2^ confidence intervals reported in Supplementary Table S2 resulted in associations between underweight BMI and identifying as bisexual in men and ‘other’ in both men and women no longer reaching significance. All other significant associations reported in Table 3 remained. Results were similar in the Natsal-3 and Understanding Society cohorts after adjustment for survey design using sampling weights. When the analysis was re-run after removing respondents with only self-reported BMI, the results were similar to the results reported above, with the following exceptions: the association between bisexual women and overweight BMI no longer reached significance (OR 1.15, 95% CI: 0.91–1.44) and the association between women identifying as ‘other’ and overweight BMI now reached significance (OR 1.48, 95% CI: 1.12–1.95). Due to very low numbers of underweight men identifying as bisexual or ‘other’, the analysis could not be re-run for these groups when self-reported weight data were removed.

## Discussion

### Main finding of this study

Our study is an important first look at the descriptive epidemiology of sexual orientation in relation to BMI categories in the UK. The results show that women who identify as lesbian or bisexual (versus heterosexual) are at increased risk of overweight or obesity, and that men who identify as gay are at decreased risk of overweight or obesity. They also indicate that women identifying as ‘other’, and men identifying as gay, bisexual or ‘other’ were at increased risk of being underweight. The associations held after adjustment for a range of covariates and were robust to several sensitivity analyses.

### What is already known on this topic

Our findings are comparable with data from the USA showing that lesbians and bisexual women tend to have a higher BMI than heterosexual women^14,34^ and gay men are more likely to have a lower BMI than heterosexual men.^20^ The differential effect of gender is an important consideration when considering health outcomes such as unhealthy weight in sexual minorities.^23^ A possible mechanism for the association of sexual orientation and BMI may be that sexual minority groups are exposed to psychosocial stressors,^50^ which may influence their health behaviours such as diet or physical activity,^51–53^ or alcohol consumption^10^ indicated in weight gain^54^ and linked to increased risk of chronic health conditions such as diabetes^24^ and cardiovascular disease.^16^

### What this study adds

The study is the first to pool population health survey data in order to consider the association between SOI and BMI in a UK population. The large LGBO sample size is a key strength of this study, allowing us to consider lesbian, gay, bisexual and ‘other’ groups separately as well as consider gender differences. Our results show that these associations can be found at the population level in the UK from representative surveys.

The large LGBO sample size also allowed us to consider the underweight category, which is frequently too small to consider in single studies and has not been included separately in previous population studies.^20,55^ Combining underweight and normal weight, commonly done in sexual minority studies,^32,33,56^ may give the impression gay and bisexual men are healthy weight. Our study, by analysing underweight separately, showed that gay men are at higher risk of being underweight.

Weight issues are inherently connected to social normative concepts of identity and desire and there are limited research that explores this in a UK context, however, international research suggests that this is a significant driver of unhealthy weight behaviours in LGB youth.^57^ Indeed evidence is available that suggests sexual minority male youth are not only more likely to engage in risky weight control behaviours than heterosexual peers and are more influenced by advertising focusing on physical appearance^58^ but also self-perceive as overweight despite being healthy or even underweight.^57^ Conversely, research suggests sexual minority women have higher levels of body satisfaction and reject the heteronormative standard of body-size, and self-perceive as being healthy or underweight when overweight/obese.^57^

The differences in weight found within the subgroups also reiterates that lesbian, gay, bisexual and those who identify as ‘other’ are not one homogenous group; gender differences are important to consider in health outcomes and in healthcare needs. The clear commitment at national and local level to address population-level weight issues provides an opportunity for policy makers and providers to use this research to better understand and address the needs of LGBO people in the UK.

### Limitations of this study

One limitation of our study is the cross-sectional nature of the surveys, so we could not consider changes in BMI or whether these associations might persist over the life course, nor their onset. Due to small numbers of respondents, we were not able to consider smaller subgroups of the non-heterosexual participants allowing us to look at the impact of intersectionality on weight in this population.^59^ Due to the small number of underweight individuals, particularly for men identifying as bisexual and men and women identifying as ‘other’, associations between underweight BMI and SOI for these groups should be interpreted cautiously. We only considered SOI in this study. Defining sexual orientation more widely (identity, behaviour and attraction) might produce different results in our sample.

It is possible that the use of self-reported weight data may have resulted in underestimation of rates of underweight, overweight and obese BMIs.^60^ Finally, we considered BMI but not other indicators of fat mass and excess weight, issues which apply to any study using BMI are unlikely to differ by sexual orientation and which have been reviewed elsewhere.^61^ Moreover, BMI is widely used in clinical and research settings to identify adults who may be at increased risk of poor health outcomes including mortality and cancer.^3,5^

Little detailed information is known about the ‘other’ category, retained by the ONS sexual orientation question^62^ and analysed in this study; a heterogeneous group, who chose to not identify as heterosexual and differ from heterosexuals on a number of variables^63^ but may experience health disparities. This group is often omitted from studies or combined with subgroups, losing data on this unique subset of the population. This selection may reflect respondents’ dissatisfaction with the current categories available for SOI^62,64^ or the lack of questions on gender identity (beyond male/female gender category) currently omitted from all UK health surveys. As it is unclear the make-up of this group, we should be cautious in the conclusions we can draw from these results.

## Conclusion

The study clearly demonstrates the link between SOI and unhealthy weight in lesbian and bisexual women and in gay and bisexual men (versus heterosexual). It is important to consider SOI health disparities in public health policy. The importance of developing tailored interventions to address these disparities and of supportive policy change to ensure development and implementation of standards of care for LGBO people are necessitated.

## Supplementary Material

final_JPH_submitted_LGBO_MIPD_BMI_paper_PRISMA_IPD_checklist_160418_fdy224

final_JPH_submitted_LGBO_MIPD_BMI_paper_STROBE_160418_fdy224

Supp_Figure_1_Women_underweight_fdy224

Supp_Figure_2_Women_overweight_fdy224

Supp_Figure_3_Men_underweight_fdy224

Supp_Figure_4_Men_overweight_fdy224

Supplementary_figures_fdy224

Supplementary_Table_1_fdy224

Supplementary_Table_2_fdy224
