# Supplementary figures and images for: Sexual orientation identity in relation to unhealthy body mass index: individual participant data meta-analysis of 93 429 individuals from 12 UK health surveys
Source: J Public Health (Oxf). 2019 Feb 21;42(1):98–106. doi: 10.1093/pubmed/fdy224 (PMC8414914; doi:10.1093/pubmed/fdy224)

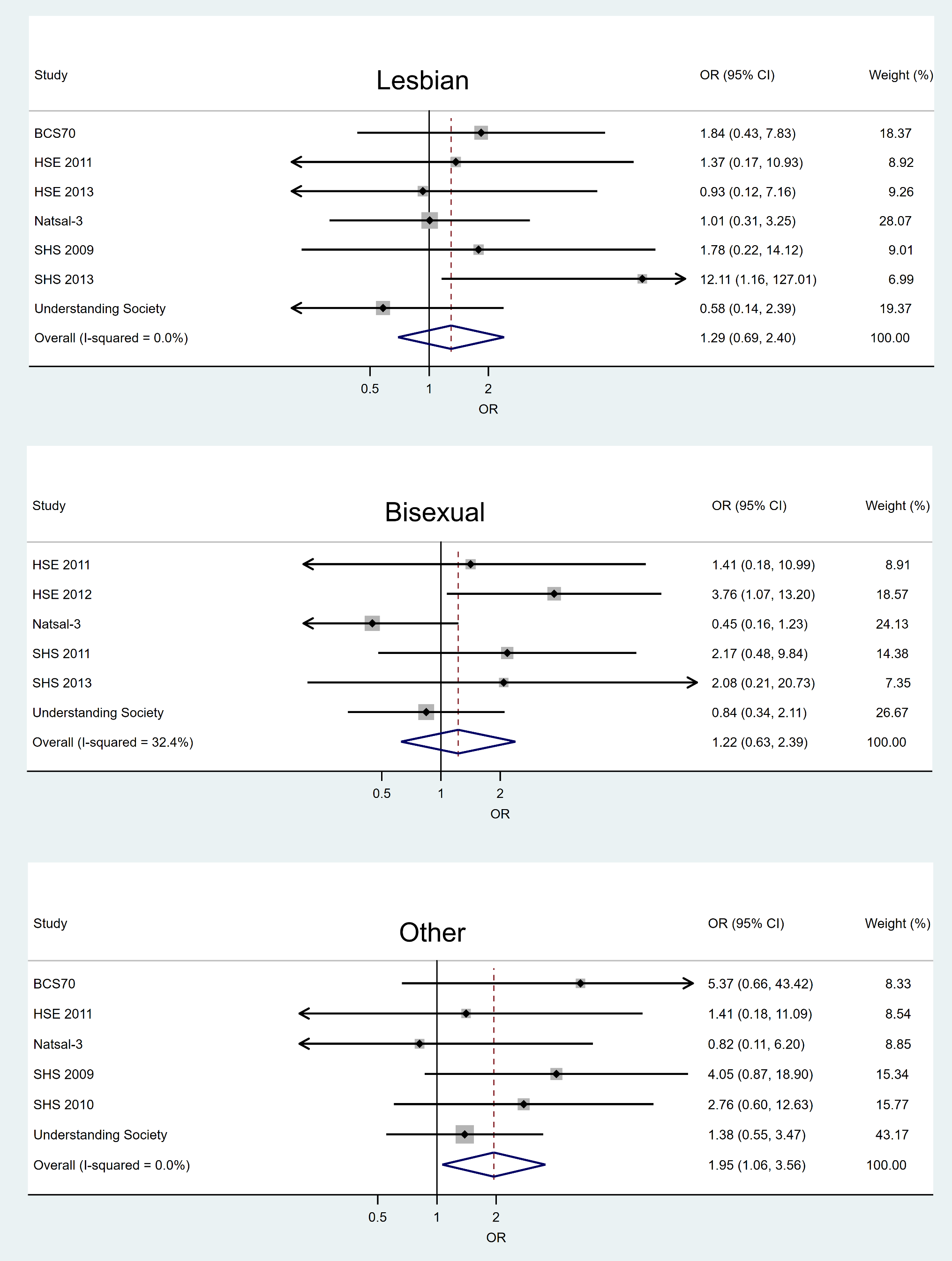

Supplement: Supp_Figure_1_Women_underweight_fdy224 [file PUBMED_42_1_98_s7.png]

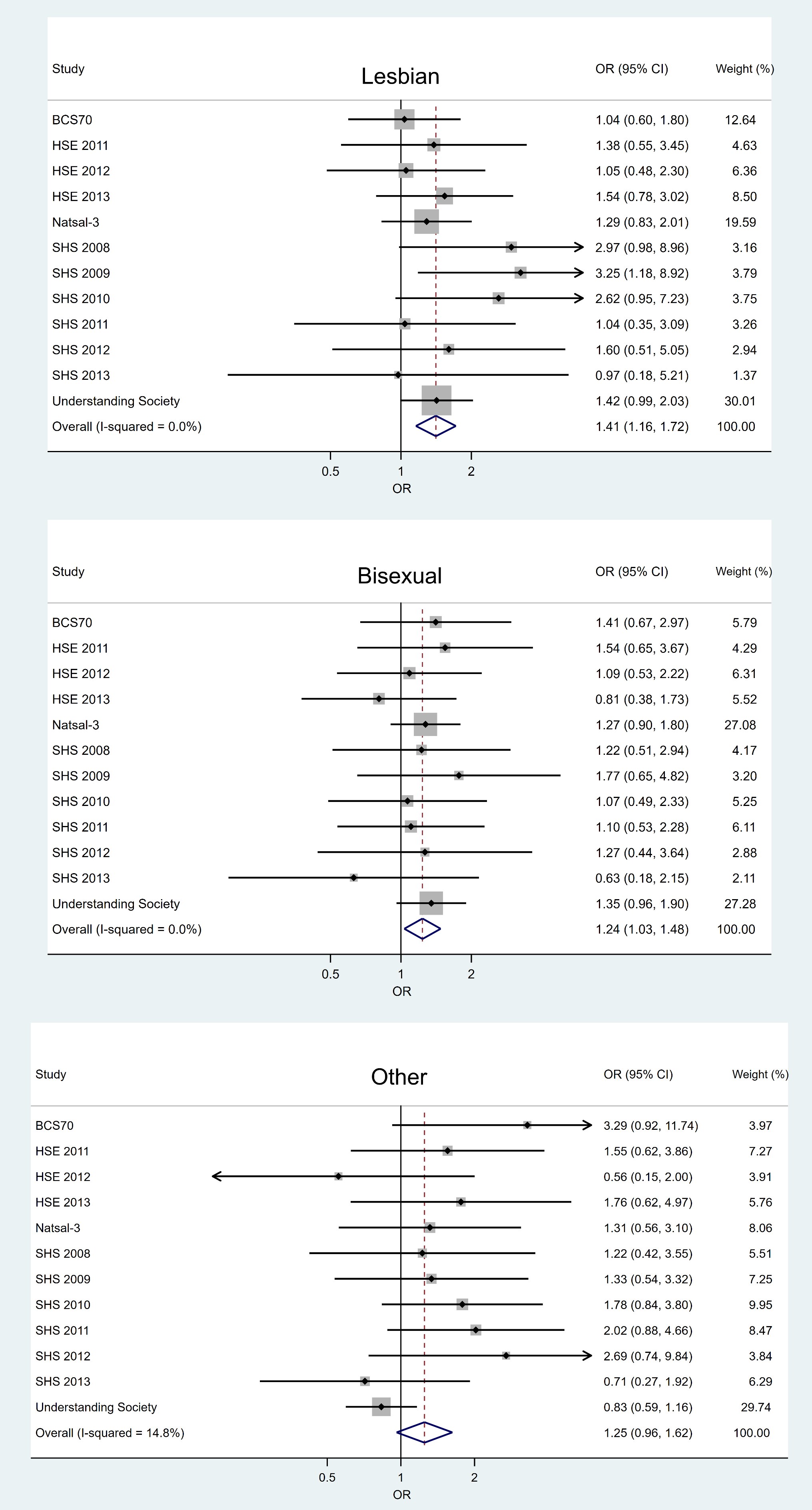

Supplement: Supp_Figure_2_Women_overweight_fdy224 [file PUBMED_42_1_98_s8.png]

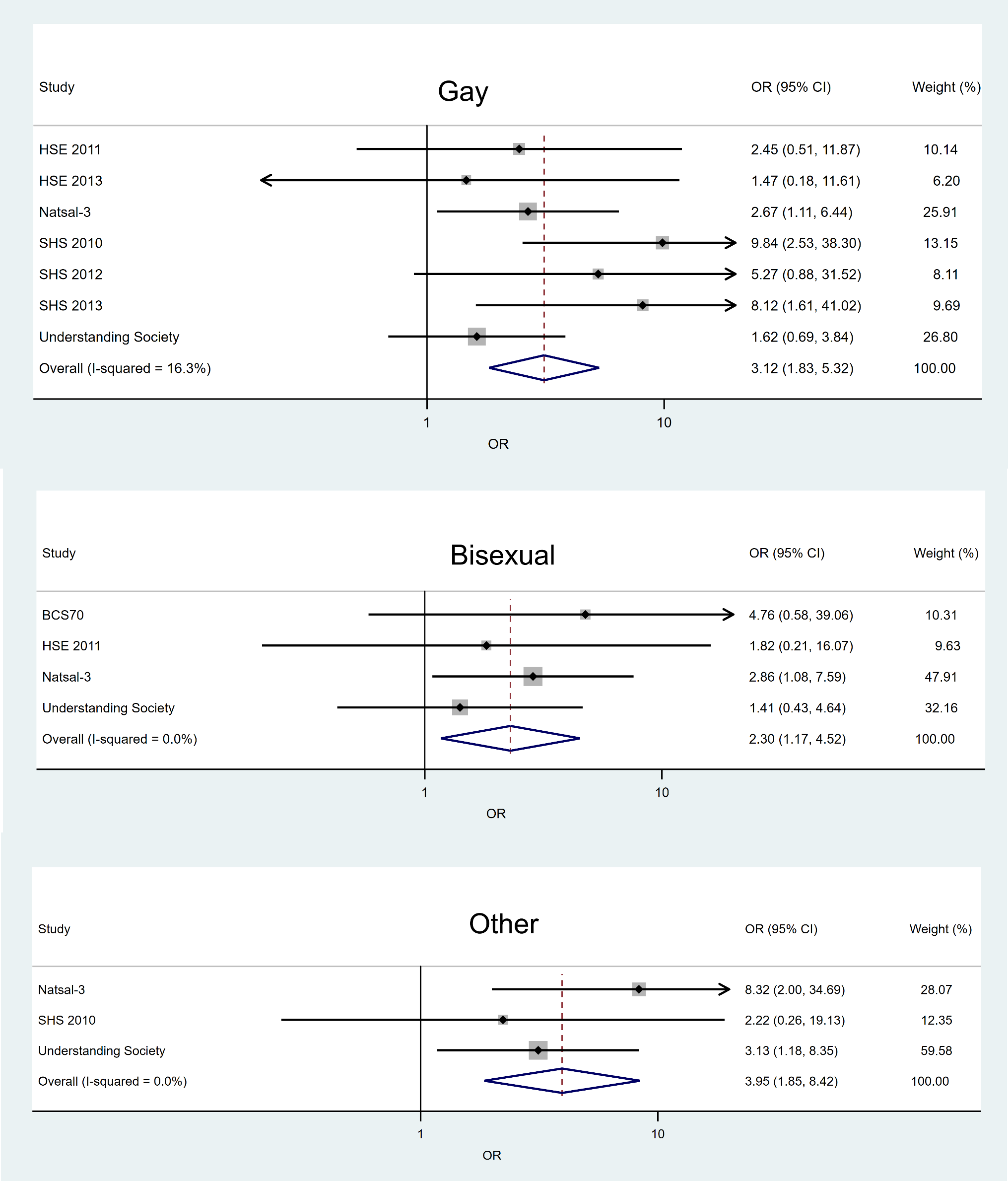

Supplement: Supp_Figure_3_Men_underweight_fdy224 [file PUBMED_42_1_98_s9.png]

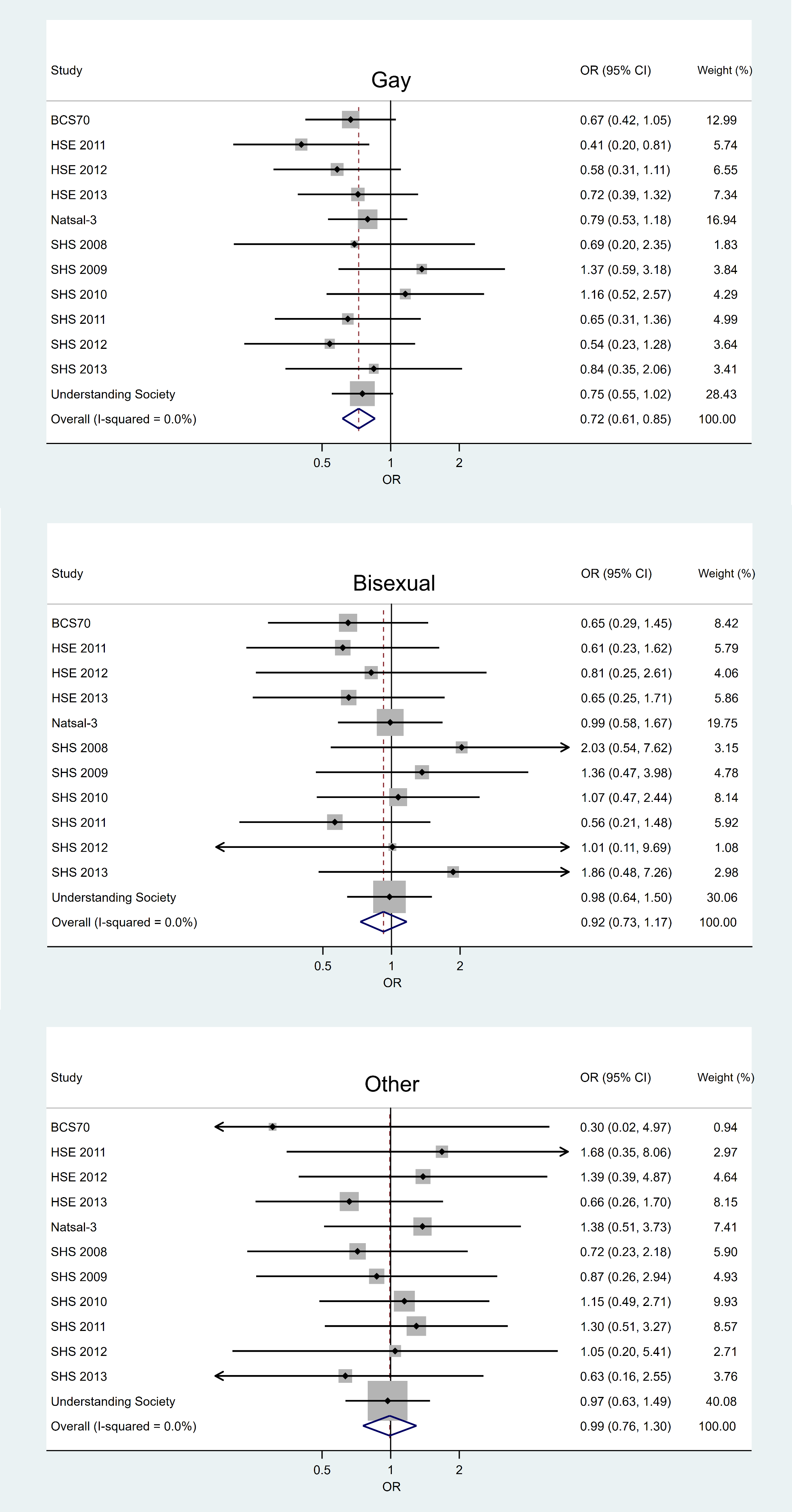

Supplement: Supp_Figure_4_Men_overweight_fdy224 [file PUBMED_42_1_98_s10.png]
