# Supplementary material for: Sexual orientation identity in relation to unhealthy body mass index: individual participant data meta-analysis of 93 429 individuals from 12 UK health surveys
Source: J Public Health (Oxf). 2019 Feb 21;42(1):98–106. doi: 10.1093/pubmed/fdy224 (PMC8414914; doi:10.1093/pubmed/fdy224)
Supplement: Supplementary_figures_fdy224 [file PUBMED_42_1_98_s4.docx]

**Supplementary figures (forest plots) - titles**

Supplementary Figure 1: Forest plots of the ORs for underweight BMI in women identifying as lesbian, bisexual or other (vs heterosexual identity), adjusting for associated covariates.

Supplementary Figure 2: Forest plots of the ORs for overweight BMI in women identifying as lesbian, bisexual or other (vs heterosexual identity), adjusting for associated covariates.

Supplementary Figure 3: Forest plots of the ORs for underweight BMI in men identifying as gay, bisexual or other (vs heterosexual identity), adjusting for associated covariates.

Supplementary Figure 4: Forest plots of the ORs for overweight BMI in men identifying as gay, bisexual or other (vs heterosexual identity), adjusting for associated covariates.
