# Supplementary material for: Sexual orientation identity in relation to unhealthy body mass index: individual participant data meta-analysis of 93 429 individuals from 12 UK health surveys
Source: J Public Health (Oxf). 2019 Feb 21;42(1):98–106. doi: 10.1093/pubmed/fdy224 (PMC8414914; doi:10.1093/pubmed/fdy224)
Supplement: Supplementary_Table_1_fdy224 [file PUBMED_42_1_98_s5.docx]

**Supplementary Table 1. Characteristics of study variables in each study separately**

| **Study name** | **British Cohort Study** | **Health Survey for England** | | | **Natsal-3** | **Scottish Health Survey** | | | | | | **Understanding Society** |
| --- | --- | --- | --- | --- | --- | --- | --- | --- | --- | --- | --- | --- |
| Study year | 2012 | 2011 | 2012 | 2013 | 2010-2012 | 2008 | 2009 | 2010 | 2011 | 2012 | 2013 | 2011-2013 |
| Study sample size | 7,551 | 6,178 | 6,159 | 6,635 | 13,806 | 4,656 | 5,463 | 5,487 | 5,545 | 3,754 | 3,829 | 24,366 |
| Refused identity item (%)^a^ | 0.5 | 3.0 | 3.0 | 2.0 | 0.4 | 6.5 | 4.6 | 5.2 | 4.4 | 2.9 | 3.1 | 0.1 |
| Underweight BMI (%) | 1.2 | 1.6 | 1.4 | 1.6 | 3.2 | 1.4 | 1.3 | 1.4 | 1.5 | 1.3 | 1.3 | 1.7 |
| Overweight or obese BMI (%) | 58.0 | 63.8 | 63.8 | 64.2 | 48.2 | 68.1 | 66.6 | 68.0 | 66.8 | 68.0 | 67.1 | 61.6 |
| Age (quartiles)^b^ | 42/42/42 | 34/47/62 | 35/49/64 | 35/48/63 | 25/34/52 | 36/48/62 | 36/47/61 | 35/49/63 | 36/49/63 | 38/50/64 | 35/48/63 | 35/48/62 |
| Male (%) | 48.0 | 45.7 | 45.9 | 46.1 | 42.1 | 44.5 | 45.5 | 44.6 | 44.5 | 45.3 | 44.0 | 43.5 |
| Lesbian/Gay (%) | 1.8 | 1.0 | 1.1 | 1.3 | 1.4 | 0.7 | 0.9 | 1.0 | 0.8 | 1.0 | 0.8 | 1.3 |
| Bisexual (%) | 0.7 | 0.7 | 0.7 | 0.7 | 1.5 | 0.9 | 0.7 | 1.1 | 1.0 | 0.6 | 0.6 | 1.0 |
| Other (%) | 0.2 | 0.6 | 0.4 | 0.6 | 0.3 | 0.7 | 0.7 | 1.4 | 1.3 | 0.6 | 0.7 | 1.0 |
| Ethnic minority^c^ (%) | 3.3 | 9.4 | 9.5 | 10.3 | 10.8 | 2.3 | 2.3 | 2.8 | 2.5 | 2.6 | 2.5 | 13.3 |
| University degree (%) | 23.8 | 25.0 | 26.3 | 26.6 | 24.9 | 25.9 | 27.4 | 28.2 | 28.0 | 29.5 | 30.1 | 25.2 |
| Smoker (%) | 24.3 | 20.2 | 18.6 | 19.2 | 27.5 | 24.9 | 24.6 | 25.2 | 23.0 | 23.7 | 21.6 | 20.8 |
| Longstanding illness (%) | 22.6 | 41.7 | 40.1 | 40.6 | 30.4 | 41.4 | 40.5 | 45.5 | 45.1 | 47.6 | 44.4 | 35.1 |
| Married/cohabiting (%) | 80.6 | 64.6 | 64.9 | 65.4 | 51.4 | 65.9 | 66.2 | 62.8 | 64.5 | 65.0 | 62.9 | 66.0 |

*Notes:* ^a^Refusal to answer sexual orientation identity item (excluded from the analytic sample). ^b^Natsal-3 over-sampled 16-30 year olds. ^c^Understanding Society over-sampled ethnic minority groups. Underweight BMI is defined as a BMI value <18.5 kg/m^2^. Overweight BMI is defined as a BMI value in the range 25 – 29.99kg/m^2^. Obese BMI is defined as a BMI value ≥30 kg/m^2^.
