# Supplementary material for: Sexual orientation identity in relation to unhealthy body mass index: individual participant data meta-analysis of 93 429 individuals from 12 UK health surveys
Source: J Public Health (Oxf). 2019 Feb 21;42(1):98–106. doi: 10.1093/pubmed/fdy224 (PMC8414914; doi:10.1093/pubmed/fdy224)
Supplement: Supplementary_Table_2_fdy224 [file PUBMED_42_1_98_s6.docx]

**Supplementary Table 2. Heterogeneity statistics for additionally adjusted meta-analyses.**

|  | **Underweight BMI** | | | **Overweight or obese BMI** | | |
| --- | --- | --- | --- | --- | --- | --- |
|  | **Cochran’s Q (degrees of freedom)** | **p** | **I^2^ (95% CI)** | **Cochran’s Q (degrees of freedom)** | **p** | **I^2^ (95% CI)** |
|  |  |  |  |  |  |  |
| **Women** | | | | | | |
| Lesbian | 5.30 (6) | 0.505 | 0.0% (0.0, 82.3) | 8.28 (11) | 0.688 | 0.0% (0.0, 65.6) |
| Bisexual | 8.19 (5) | 0.146 | 32.4% (0.0, 87.1) | 3.87 (11) | 0.974 | 0.0% (0.0, 1.9) |
| Other | 3.31 (5) | 0.653 | 0.0% (0.0, 77.7) | 14.32 (11) | 0.216 | 14.8% (0.0, 72.5) |
| **Men** | | | | | | |
| Gay | 7.32 (6) | 0.292 | 16.3% (0.0, 83.6) | 7.74 (11) | 0.737 | 0.0% (0.0, 61.7) |
| Bisexual | 1.34 (3) | 0.719 | 0.0% (0.0, 85.1) | 6.16 (11) | 0.863 | 0.0% (0.0, 48.7) |
| Other | 1.53 (2) | 0.464 | 0.0% (0.0, 97.0) | 3.76 (11) | 0.976 | 0.0% (0.0, 6.4) |

*Notes:* Cochran’s Q score is a measure of variation in outcome estimates across studies. A significant Cochran’s Q score (p<0.05) indicates that there is more variation in the data than would be expected as a result of sampling error alone. I^2^ estimates the proportion of total variation in the data due additionally to heterogeneity in the true effects across studies.
